# Exploratory Study: The Impact of Online Coordinative Exercise in a Small Latinx Youth Sample

**DOI:** 10.3390/pediatric18010013

**Published:** 2026-01-19

**Authors:** Nancy J. Hernandez, John S. Carlson

**Affiliations:** Department of Counseling, Educational Psychology & Special Education, College of Education, Michigan State University, East Lansing, MI 48824, USA; herna775@msu.edu

**Keywords:** executive functioning, inattention, hyperactivity, intervention, physical activity, Latinx youth

## Abstract

**Background/Objectives:** The effects of online physical activity (PA) interventions on executive function (EF) and Attention-Deficit Hyperactivity Disorder (ADHD) symptoms are promising; nonetheless, their benefits for Latinx youth remain unclear. **Methods:** This study explores levels of adherence, cognitive and behavioral outcomes and acceptability of an online PA intervention, Zing Performance, among a Latinx youth sample; only a few of the participants completed their condition (*n* = 6). **Results**: There was wide variability in adherence levels at mid-treatment (*n* = 5) and high-level adherence at post-treatment (*n* = 2). A Mann–Whitney test yielded a statistically significant (*p* = 0.004) improvement in the treatment group’s inattention symptoms at mid-treatment (*n* = 5), compared to the Waitlist Control; (WLC; *n* = 6). EF and hyperactivity/impulsivity were not significantly different. Further, pre-, mid- and post-participant trajectory data revealed that one participant benefited significantly from treatment, one participant demonstrated little to no response to treatment, and most of the WLC participants remained in the severity ranges throughout the 12 weeks. The parents of the two children who completed treatment reported high levels of acceptability informally and on the quantitative measure. **Conclusions:** Exploratory findings support further investigation of Zing among Latinx families with cultural consideration to study procedures. The lessons learned from this study are valuable for future research procedures and interventions with this marginalized population.

## 1. Introduction

### 1.1. Cognitive and Behavioral Symptomology

Executive functions (EFs) are goal-directed skills made up of three cognitive core functions: working memory (WM), inhibition and cognitive flexibility [1]. Deficits in EFs correlate with deficits in attention, planning and problem-solving [1,2]. These attention-deficit hyperactivity disorder (ADHD)-related domains activate the brain’s frontal region, which strongly influences EF (i.e., inhibitory control and attention) [1,2]. Researchers have hypothesized that decreased activation of the cerebellar-frontal neural circuits is directly linked to hyperactivity and impulsivity symptoms [3]. Hence, the evolving literature [4] has evaluated the effects of physical activity (PA) and associated brain activation with cognitive (i.e., executive functioning) and behavioral (i.e., hyperactivity/impulsivity) domains in children who exhibit attention and hyperactivity/impulsivity challenges. Researchers have proposed PA as a promising complementary or alternative treatment for those with ADHD due to its positive cognitive and behavioral outcomes [4].

### 1.2. Physical Activity as an Evidence-Based Practice for Improving Executive Functioning

It is essential to evaluate the current state of the PA intervention literature using an evidence-based practice lens to assess intervention effectiveness and consumer values [4]. PA research consistently evaluates treatment effectiveness on children with attention and hyperactivity/impulsivity challenges [5]. Nonetheless, these studies regularly overlook critical components of evidence-based practice, such as treatment adherence and treatment acceptability, which are critical components to improving and sustaining the care of all in need of services [4]. Adherence and acceptability are imperative within the provision of treatment to families of children with EF, attention and hyperactivity/impulsivity deficits as parents tend to support intervention completion rates when they perceive them to be efficient and effective in addressing their child’s behavioral health needs [6,7].

In considering treatment sustainability components, such as treatment adherence and treatment acceptability, it is critical to examine the specific nature of the PAs that have been investigated in those demonstrating behavioral health challenges [4,5]. The literature considers PA to be any bodily movement that produces caloric expenditure by one’s skeletal muscles [8]. Due to the broad definition, multiple PA types exist, such as aerobics, coordinative exercise and combination or mixed [6]. Aerobic exercise involves walking, jogging, or biking [9], while coordinative exercise stimulates an individual’s brain to conduct sequenced cognitive and motor activity [6]. A combined approach often involves a mix of aerobic and coordinative exercise components [10,11,12]. PA interventions typically vary widely in duration (e.g., 1–30 min), frequency (e.g., 2–5 times a week) and implementer (e.g., computer/system or trainer/researcher) [8,10]. PA variations explored in the literature have resulted in mixed findings on children’s cognitive and behavioral outcomes [5,6]. Hence, due to procedural inconsistencies among research studies, the effect of exercise type moderators (e.g., aerobic vs. coordinative exercise vs. aerobic/coordinative exercise; duration) on cognition and behavior remains a crucial gap in the literature to inform evidence-based practice.

PA has been studied across non-clinical and clinical samples [3,5,11]. Findings from a recent meta-analysis support PA’s positive benefit for children with both parent-reported ADHD symptoms and formal ADHD diagnoses [6]. This study highlighted the findings of 15 randomized experimental trials (e.g., control, crossover) and two quasi-experimental studies that focused on multi-week PA’s influence on mental health outcomes in children with ADHD and/or Oppositional Defiance Disorder (ODD). Most of the studies focused on targeting change in both cognition and motor skills. Independent variables included a range of 10 different PA types (e.g., exergaming, table tennis, physical cognitive training) and were implemented across multiple weeks, ranging from four to eighteen weeks. Small-to-moderate reductions in parent-rated inattention and hyperactivity/impulsivity symptoms were reported, suggesting promising outcomes for cognition- and motor skills-focused PA. Nevertheless, effectiveness evaluations differed substantially across studies, potentially due to their varied procedures and designs, such as a wide range of multi-week durations and limited studies including a control group, while others integrated a comparison. These two methodological factors serve as limitations and demonstrate the importance of investigating the moderating effect of duration and the use of rigorous experimental trials. A promising implication from this meta-analytic investigation was that parents conceptualized PA as a better medium to improve behaviors than ADHD medication, supporting the need to explore treatment acceptability in future PA studies. Neudecker et al. [5] reported that adherence varies widely between studies, yet they fail to specify if it is within the acceptable range (i.e., 80–100%). As adherence is inconsistently or vaguely reported on [6], the relationship between PA type, duration, adherence, effectiveness and acceptability remains unknown.

### 1.3. Aerobic and Coordinative Exercise

Aerobic PA studies involving exercises of varying types and durations have yielded mixed results for improving cognitive and behavioral outcomes [8,9]. Aerobic study findings range from no statistically significant effects to statistically significant moderate improvement in inattention (*d* = 0.69) and hyperactivity (*d* = 0.65) symptoms within school-aged populations based on parent reports [11,12]. Studies investigating PA involving coordinative exercise on children’s cognitive and behavioral outcomes are generally more effective compared to aerobic exercise [13,14,15]. Moreover, children with ADHD may benefit from cognitively challenging exercises like those associated with coordinative exercises and routines [5,6,13]. However, studies demonstrating the effectiveness of coordinative exercise have yet to formally report adherence or acceptability outcomes. Such outcomes, combined with effectiveness data, are essential to generalize the impact of this type of PA on more diverse groups of children experiencing deficits in EF or ADHD symptoms [4,10].

Neudecker et al. [5] summarized PA interventions into one of four categories––acute running or cycling, mixed long-term, specific and sensori- or perceptual-motor training exercise types. Mixed exercise (e.g., running, coordination tasks) yielded the most favorable results on cognitive and behavioral outcomes, further supporting the benefit of coordinative exercise for school-aged children. The studies involved in this review, however, also failed to evaluate treatment adherence or acceptability. Therefore, even though mixed exercise programs appear promising, their true clinical utility remains undetermined without further study [7,16,17].

### 1.4. Online Coordinative Exercise

Online PA interventions often include both the combination types (e.g., aerobic and coordinative exercise), with the primary foci being balance and coordination for children with cognitive and behavioral challenges [5,15,17]. Ji et al. [13] demonstrated evidence of coordinative exercise brain stimulation, leading to their hypothesis that coordinative exercise may contribute more to the positive cognitive effects when compared to aerobic PA. These interventions continue to be studied because of their significant impact on children with cognitive and behavioral challenges [5,6].

Few published studies have exclusively investigated coordinative exercise compared to a different PA intervention or a control group [4,18,19,20,21]. Coordinative exercise studies are novel and in need of further investigation within an evidence-based practice lens among children with cognitive and behavioral challenges [7]. Ziereis and Jansen’s [16] work contributed to the coordinative exercise literature, where participants demonstrated significant changes in cognitive domains compared to the control group. Nevertheless, the coordinative exercise research is limited and warrants further study.

When establishing an evidence-based intervention, it is important to consider potential accessibility issues and other barriers to treatment, as marginalized populations (e.g., Latinx) often encounter difficulties with access (e.g., related to transportation, distance to care, community resources) [21]. Therefore, coordinative exercise programs facilitate access through online formats for some populations [20,21]. Therefore, online coordinative exercise warrants further study due to its potential to support treatment access and promote treatment completion. In addition, online intervention can ease replicability, potentially improving treatment adherence rates due to automated systems [3,4,18,19]. Lastly, online interventions may be appealing due to the speedy dissemination of automated feedback to the client and families through self-monitoring tools and progress reports.

The critical component among online PA interventions is the coordinative exercise element [13]. Online coordinative exercise programs have been developed based on the available evidence that coordinative exercise may improve cognitive and behavioral symptoms [5,6]. One intervention, Zing Performance (Zing), was created and uses research data to further modify its program to help various age groups [22]. The initial research on Zing was promising in older adults and adolescents who were at-risk of school dropout [18,19,22]; however, research remains extremely limited for the population of Latinx children with ADHD. Given our target population and the gaps this study aims to address, it is important to disseminate the results of those who participated in the current initiative [4]. The current study sought to address the gaps in the literature by emphasizing this underrepresented population and the 12-week coordinative exercise treatment’s adherence, impact and acceptability of Zing. It was hypothesized that those who engaged with treatment would adhere to the intervention as intended and would show small nonsignificant improvements at mid-treatment and low levels of cognition and behavioral severity at post-treatment compared to the control group, and parents would rate Zing as acceptable (i.e., Treatment Evaluation Inventory-Short Form; TEI-SF; score of 31 or higher).

## 2. Materials and Methods

### 2.1. Participants

**Figure 1:**
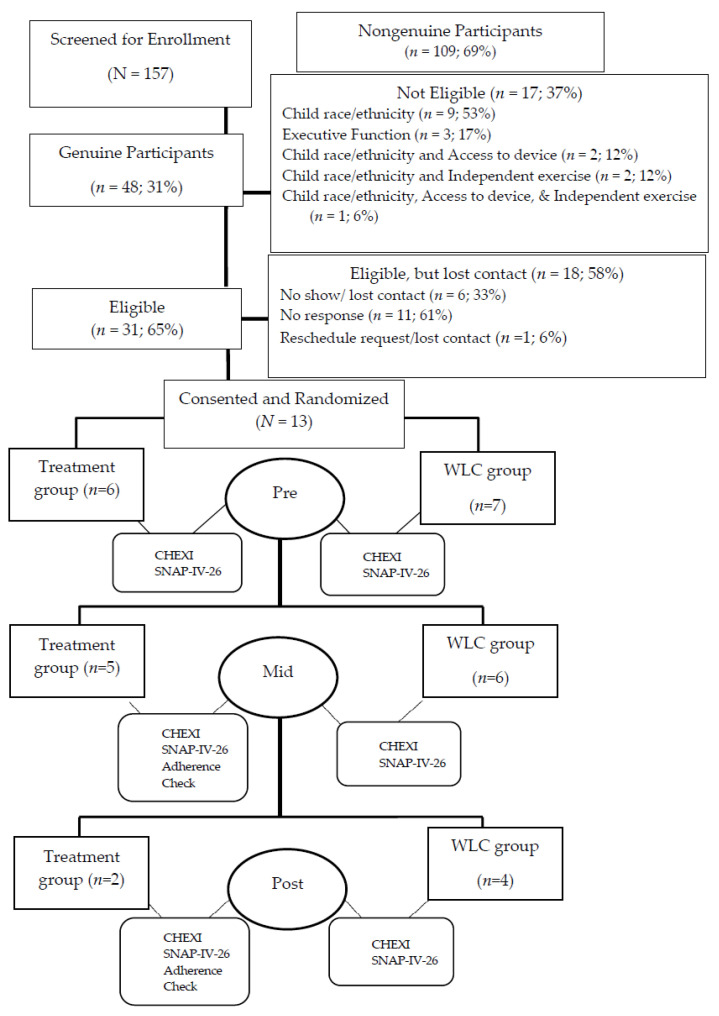
Intervention trial flowchart.

One hundred fifty-seven participants were screened for study enrollment via a comprehensive web-based recruitment platform (i.e., Qualtrics Survey tool) accessed through Michigan State University in East Lansing, Michigan. Screening took place from December 2023 to August 2024 (see Figure 1). Non-genuine participants (i.e., bots; *n* = 109) were detected by cluster enrollment and inconsistent geographic data [23]. The inclusion criteria consisted of the following: (a) children ages 7 to 11 years; (b) identify as Latinx; (c) parent-reported mild or more significant challenges in one or more of the following areas: WM, inhibition, inattention, or hyperactivity/impulsivity; (d) parents or legal guardians having the ability to complete the measures in English; (e) parents or legal guardians endorsing that their child could independently perform non-rigorous PA; and (f) access to an electronic device with the internet to complete the Zing Performance program. There were no inquiries on concomitant treatments on the screener.

Thirteen dyads consented to participate in the originally designed, randomized control trial and enrolled in their randomly assigned conditions (i.e., treatment or WLC) on a rolling basis. The researchers encountered the difficult decision to move forward with a revised methodology of a small exploratory trial with a sample of 13. Ultimately, the researchers made the decision to continue due to the persistent challenges of unsuccessful communication and coordination efforts with the other 18 eligible dyads. The first author’s graduate school timeline for research requirements justified the necessity of this change. Six of the thirteen assenting children were randomly assigned to the treatment condition and the other seven to the WLC condition. All dyads involved a maternal caregiver. The average age of children in the treatment group was 8.2 years, comparable to 8.3 years for the WLC group. Similarly, gender was comparable across groups, with two girls in the treatment group and three girls in the WLC group. The demographic information collected was aligned with the study’s inclusion criteria. Therefore, no demographic variables outside of age, gender, child ethnicity, parent relationship and their ethnicity were collected; other comparisons between the two groups can be found in Table 1. Eleven of the thirteen completed half of their assigned condition, allowing for an exploratory analysis on the impact of treatment from pre- to mid-treatment. Further, only six of the original thirteen participants completed the entirety of their 12-week condition: two children in the treatment group and four in the WLC.

### 2.2. Study Design and Procedures

Initial recruitment and study enrollment challenges required a number of study modifications because the initial recruitment flyer did not yield any responses. Initially, the physical and e-flyers, which contained the study’s brief (i.e., ≤15 min to complete) Qualtrics screener link and information, were shared with Latinx and community-based organizations, schools (e.g., elementary and middle schools) and through social media posting in parenting and mental health professional groups and personal networks. After consultation with the Latinx community, a set of revisions was made to successfully reach targeted families. For example, to increase accessibility, both the QR code and the link were shared with the study information. In addition, greater emphasis was placed on the value of the online intervention (i.e., USD 500) that was being offered for free and the availability of small monetary (i.e., USD 50) remuneration for study involvement. A total of 31 eligible participants, just above the targeted number of 30, were invited to participate. Once someone was deemed eligible, the first author invited participants to a virtual meeting to review the consent and assent forms. Most of the email invitations went unanswered, despite three follow-up emails including incentive reminders. Notably, only 13 of the eligible 31 dyads agreed to schedule a time to meet with the first author to review the study procedures (e.g., study procedures, intervention implementation, data collection) and all confirmed participation via the consent/assent forms. At the initial meeting, most of the 13 eligible mothers reported that they had reviewed the consent form before the meeting and were ready to sign. Nonetheless, the first author followed study procedures and reviewed the consent and assent forms with the parent and the child to ensure comprehension of all study expectations. The first author randomized ID numbers, using a Random.org web-generator, before participants were invited to meet with her virtually. After the consents and assents were signed at the virtual meeting, the first author assigned the participant the next available ID using the pre-made conditions. Although both groups engaged with the treatment at different timepoints of the data collection, parents from both groups experienced Wi-Fi difficulties when setting up the intervention on their technological devices. These difficulties delayed the application download process, extending the virtual call. To attempt to download the application rapidly, parents intended to problem-solve and momentarily turned their videos off during the virtual call, asked people in the household to disconnect from Wi-Fi and attempted to download it on separate electronic devices.

Those who were assigned to the treatment group completed the Zing program, which was composed of two 10–15 min exercise sessions per day for 12 weeks. The first author asked those participants in the treatment group to download the application to support participants through the account set-up, logging in and navigating the application. Data collection included adherence checks at mid- and post-treatment, collected parent and child-outcome measures at pre-, mid- and post-treatment, and then collected parent acceptability data at post-treatment. Zing’s novel coordinative exercise online approach has the potential for children to improve EF and their inattention and hyperactivity/impulsivity symptoms [4,5,6]. An example of a Zing coordinative exercise consists of standing up on one leg for a few seconds and keeping balance; activities become more challenging by having to close one’s eyes and balance on one leg. The constant repetition of exercise helps participants develop skills designed to make activities effortless. Coordinative exercise is posited to stimulate the cerebellum, enhancing the brain’s efficiency in processing information. Participants’ individual progress was tracked through program-embedded neurological assessments (e.g., evaluating response time, reading) at the end of each month [22,24]. No parameters or data collection procedures were established regarding external treatment (e.g., medication treatment, behavior therapy, other recreational activities) engagement; therefore, this data is not available.

Those who were assigned to the WLC group completed outcome measures throughout their waitlist period (i.e., pre-, mid- and post-) and gained access to the intervention after completing post-measures. Similarly to the treatment group, the WLC group also received application set-up support. No parameters were set and no information was collected pertaining to adjunctive treatments. Nevertheless, one of the parents of a child in the WLC group shared that her child was on medication at the start of the study, hoping it would not exclude her from participating in the study. She was relieved that it was not an exclusionary factor but there was no further information on the child’s medication care shared or inquired about.

### 2.3. Measures

Treatment adherence within this study was an average percentage of weekly sessions completed at 6 weeks (mid-treatment) and 12 weeks (post-treatment). The Zing program monitors treatment adherence per user, expecting them to complete two sessions a day for a total of 12 weeks (i.e., 14 sessions per week × 12 weeks = 168 sessions). The researchers used the 80% adherence (135 out of 168 sessions) research standard to help inform the effectiveness of treatment [10]. Therefore, participants were expected to have high-level adherence by completing at least 80% of their 10–15 min weekly exercise sessions to comply with the treatment (i.e., a maximum of 168 sessions were available) at mid- and post-treatment. The program integrates weekly summaries of sessions completed that were emailed to the participant’s caregiver or legal guardian. For instance, if a user completed 14/14, 10–15 min sessions for a given week, they would have met full (100%) adherence to the Zing program that week.

Cognition Outcomes. Working Memory and Inhibition. The Childhood Executive Functioning Inventory (CHEXI) [25,26] is a 24-item parent form that uses a Likert scale from one (definitely not true) to five (definitely true). The CHEXI is for children 4 to 12 years old and assesses EF in two main functions—WM and inhibition. The WM and the inhibition index are composed of two subscales, each with good test–retest reliability, respectively: WM (*r* = 0.75), planning (*r* = 0.94), regulation (*r* = 0.84) and inhibition (*r* = 0.86) subscales. Samples have been tested to indicate cutoff scores for WM and inhibition in clinical ADHD populations. Inclusion criterion scores for this study were lower (i.e., indicating fewer challenges) than the scores reported in the literature because the researchers were not recruiting a clinical sample, while published studies focused on clinical symptoms [25,26,27].

Inclusion criteria were met with mild elevation scores in at least one of the EF indexes—WM and inhibition of 30 and 26 or higher, respectively. This measure was intended to differentiate cognitive (i.e., EF) from behavioral (ADHD symptoms) characteristics. It previously demonstrated statistically significant low to moderate criterion validity (*r* = 0.19– 0.39) and showed good overall model fit [χ^2^ (296) = 686.17, *p* < 0.001, RMSEA = 0.04, CFI = 0.95, SRMR = 0.04] for a two-factor model [27,28]. Additionally, the CHEXI score’s test–retest reliability was found to be adequate for research purposes (*r* = 0.89, *p* < 0.001), and the subscales were also in the reliable range with no significant difference between the test–retest data points—WM (*r* = 0.75), planning (*r* = 0.94), regulation (*r* = 0.84) and inhibition (*r* = 0.86) [28]. The CHEXI reliably gauged WM and inhibition progress.

Behavioral Outcomes. Inattention and Hyperactivity/Impulsivity. The MTA Swanson, Nolan and Pelham-IV (MTA SNAP-IV) is a revised and reduced version of the SNAP-IV [29,30,31] and was used to evaluate inattention and hyperactivity/impulsivity progress. The MTA SNAP-IV is a 26-item scale completed by the parent. Each item is rated using a four-point scale ranging from 0 (not at all) to 3 (very much). Items are grouped into three subscales––inattention (9 items), hyperactivity/impulsivity (9 items) and opposition/defiance (8 items). However, only the first two subscales were used for the study. A score of 13 indicates mild symptoms for inattention and hyperactivity/impulsivity subscales, and an increasing score indicates more challenges. The subscales align with DSM-IV criteria for ADHD and ODD. The coefficient alpha for the overall parent rating reliability is 0.94 [29]. Specifically, for inattentive it is 0.90 and for hyperactivity/impulsivity it is 0.79. Lastly, a three-factor confirmatory analysis suggests a strong goodness-of-fit (0.99) for the SNAP-IV parent model.

Treatment Acceptability. The TEI-SF is a 9-item, 5-point Likert scale ranging from 1 (strongly disagree) to 5 (strongly agree) [32]. The TEI-SF gauges the level of perceived treatment acceptability. Scores on the TEI-SF range from 9 (low acceptability of treatment) to 45 (high acceptability of treatment), with a cutoff score of 27 representing moderate acceptability [32]. The TEI-SF has a coefficient alpha of 0.85, which is acceptable for research purposes. Therefore, the TEI-SF measure was used to inform treatment acceptability for this study because of its acceptable psychometrics. In addition to quantitative measures, some parents reported informal treatment acceptability data, which is reported in our exploratory analysis.

### 2.4. Analysis

An intent-to-treat analysis was originally planned to evaluate the impact of Zing between those who received treatment and those in the WLC. Unfortunately, due to our high noncompletion rate, statistical constraints made that impractical. Considering the variation in treatment duration that currently exists in the literature [5,6], analyzing the treatment impact on this small sample was deemed crucial. The researchers conducted a bootstrapping test that was deemed inconclusive due to the small pre- and post-treatment samples, despite the use of 500 simulations. Therefore, the Mann–Whitney *U* test was used to analyze the treatment impact on the sample (*n* = 11) at the 6-week timepoint. To further understand the individual pre-, mid- and post-treatment trajectories for those who completed their conditions (*n* = 6), the raw scores for each outcome measure are reported.

Mann–Whitney *U* Test. The Mann–Whitney statistical analysis provided insight into the between-group outcomes at 6 weeks in the 11 participants who completed at least half of their condition. Comparable sample sizes between groups (treatment *n* = 5, WLC *n* = 6) were used for a meaningful analysis, despite the small sample sizes. The Mann–Whitney test compares the distribution of two independent samples, which is useful to analyze effects in a small sample [33]. This test supports the researchers’ ability to identify potential differences between the two groups and gain valuable insights about how participants responded to treatment compared to those in the WLC group.

## 3. Results

### 3.1. Treatment Adherence

Adherence (i.e., of completed 10–15 min exercise sessions completed at six weeks and twelve weeks; two sessions expected per day) was monitored for those who were assigned to the treatment condition at mid- (i.e., 6 weeks) and post-treatment (i.e., 12 weeks). The five participants demonstrated a wide range (44–95%) of treatment adherence. Notably, those who dropped out before completing the treatment had lower and more variable in-session completion rates (i.e., 44%, 50%, 68%) compared to those who completed treatment (i.e., 85, 95%) at the 6-week timepoint. Parents that were in the lower end of treatment adherence rates attributed the lack of exercise completion due to family illness (e.g., family members following a domino effect and getting sick at different times, affecting parent supervision availability for intervention), losing Wi-Fi due to natural disasters (e.g., hurricane/storm damage) and balancing the exercises with after-school activities (e.g., guitar lessons). However, the two parents of those who completed treatment also reported some illness in the family, extracurricular activity involvement and intermittently limited parent availability due to extended work hours. Their children demonstrated above-acceptable rates of adherence. Specifically, at mid-treatment, they completed 85% and 95% of their total expected sessions, respectively. By post-treatment, the participants completed 81% and 96% of their sessions, achieving high-level adherence, meaning that this treatment group adhered to Zing as intended.

### 3.2. Cognitive and Behavioral Outcomes

Pre- to Mid-Treatment. The Mann–Whitney *U* test yielded valuable inferences on the pre- to mid-treatment between-group differences (*N* = 11). Specifically, those who engaged in 6 weeks of Zing (*n* = 5) demonstrated statistically significant differences in inattention (*U* = 0, *p* = 0.004; Table 2) compared to the control (*n* = 6). No statistical differences were found between the treatment and the WLC groups for WM (*U* = 6, *p* = 0.06), inhibition (*U* = 6.5, *p* = 0.07) or hyperactivity/impulsivity (*U* = 6.5, *p* = 0.07).

**Figure 2:**
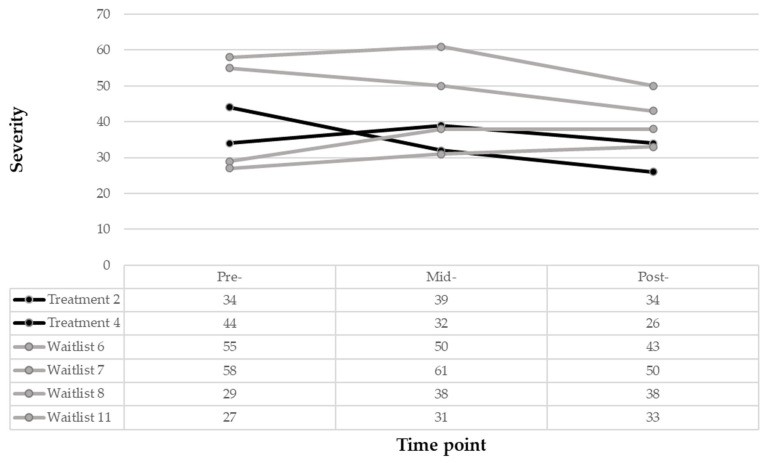
Working memory treatment and waitlist participant trajectories.

Pre-, Mid- and Post-Treatment. It is beneficial to note the different symptomatic profiles at the individual level due to the small sample size of this exploratory study. The following explores the symptomatic profiles of the two children who completed the treatment (i.e., treatment 2 and 4) and of the four children who completed the waitlist condition (i.e., treatment 6, 7, 8, 11). As seen in Figure 2, at pre-treatment, both treatment children demonstrated mild (raw score of 34) to moderate (raw score of 44) WM difficulties, and those in the WLC demonstrated either mild (raw scores of 27, 29) or severe (raw scores of 55 and 58) WM severity. It is important to note that only one participant (treatment child 4) in this sample obtained a level of not clinically significant WM at 12 weeks, which was observed in the treatment group (raw score of 26). Notably, treatment child 4 demonstrated an improvement at six weeks by scoring at a mild level (raw score of 32) of WM. Waitlist children 8 and 11 had less severe WM levels at pre-treatment onset, demonstrated worsened severity at mid-treatment (raw scores increased 4 and 9 digits, respectively) and demonstrated minimal changes at post-treatment (raw scores changed 0 and increased 2 digits, respectively). Similarly, waitlist children 6 and 7 had higher severity (raw scores of 55 and 58, respectively), and one improved at mid-treatment (raw score changed 12 digits) while the other worsened (raw score changed 5 digits).

**Figure 3:**
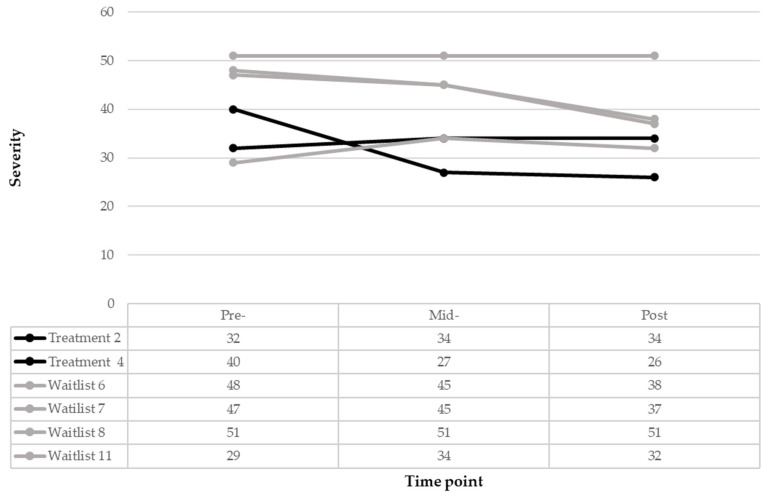
Inhibition treatment and waitlist participant trajectories.

Figure 3 demonstrates the participant-level trajectories of the six participants who completed the six weeks of their condition. Treatment children 2 and 4 demonstrated opposite responses to treatment at six weeks (increased 2 digits and decreased 13 digits, respectively). At post-treatment, treatment child 2 remained at the same level (raw score of 34), and treatment child 4 reached not clinically significant inhibition levels (raw score of 26), again being the only one in the sample to achieve this improvement. Waitlist child 11 demonstrated similar mild levels of change compared to treatment child 2, with worsened severity at mid-treatment (change score of 5) and slightly improved (change score of 2) at post-treatment. Whereas two other participants in the WLC group demonstrated severe inhibition levels at treatment onset (raw scores of 47 and 48, respectively), then slightly improved and scored at the moderate levels at 6 weeks (raw scores of 45) and 12 weeks (raw scores of 38 and 37). Waitlist child 8 remained at the mild inhibition (raw scores of 51) severity level at pre-, mid- and post-treatment.

**Figure 4:**
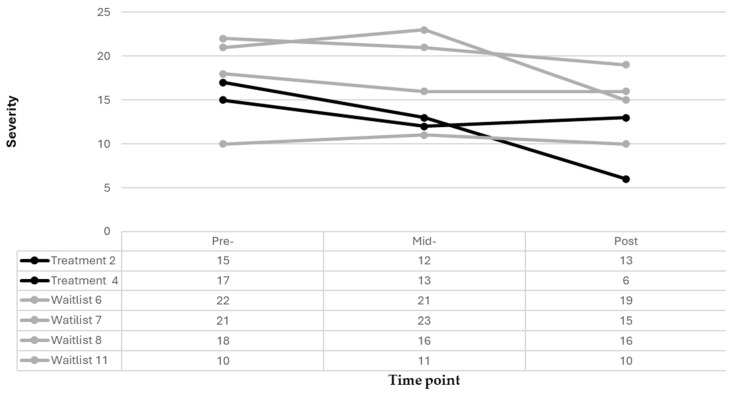
Inattention treatment and waitlist participant trajectories.

There were slight improvements in inattention at the six-week timepoint for most of the participants in this sample, as evidenced by a decrease in raw scores for four out of the six participants, despite their condition (Figure 4). Nevertheless, it is notable that one participant in the treatment group had a mild inattention score (raw score of 15) at treatment onset and scored in the not clinically significant (raw score of 12) range at six weeks. However, this participant (treatment child 2) scored in the mild range (raw score of 13) for inattention at 12 weeks. Only one participant in this sample demonstrated a significant improvement (change score 4 and 7, respectively) in inattention at mid- and post-treatment and they received the full intervention.

**Figure 5:**
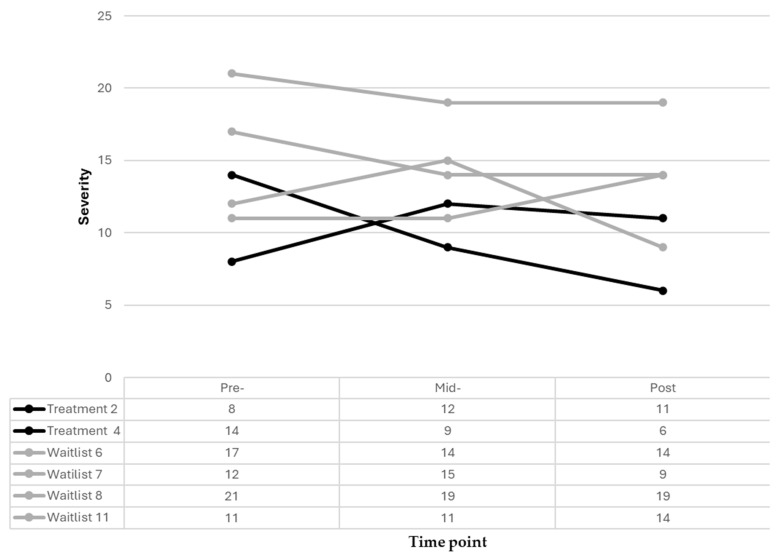
Hyperactivity/impulsivity treatment and waitlist participant trajectories.

Of the six participants who completed their conditions, no one had scored at severe hyperactivity levels at treatment onset (Figure 5). Both groups obtained one participant who started and progressed with not clinically significant levels of hyperactivity/impulsivity at treatment onset (raw scores of 8 and 11) and at mid-treatment (raw scores of 12 and 11). Notably, the participant in the treatment group (treatment child 2) remained with not clinically significant levels (raw score of 11) of hyperactivity/impulsivity at 12 weeks, while the participant in the WLC scored mild levels (raw score of 14) of hyperactivity/impulsivity at the 12-week data collection. Similarly, both participants in the treatment group ended treatment with not clinically significant levels, while the treatment group’s children ranged from not clinically significant (*n* = 1), mild (*n* = 2) and moderate (*n* = 1) levels of hyperactivity/impulsivity.

### 3.3. Treatment Acceptability

Acceptability data were only formally collected for those who completed the treatment condition. As hypothesized, the two parent participants who completed Zing found it to be highly acceptable and well above the criterion of 31, with scores of 36 and 43 [32]. Parents rated items like “I believe this treatment is likely to be effective” and “Overall, I have a positive reaction to this treatment” with the highest score of five points or “strongly agree.” Although treatment acceptability was not formally collected from the children, the parents reported that one child had mixed feelings about the exercises; she enjoyed some, but not all. In addition, the other child enjoyed and was self-motivated to complete his daily exercises.

## 4. Discussion

The current exploratory study provides an initial evaluation of the impact of coordinative exercise on a small sample of Latinx youth who presented with mild or more severe EF and/or ADHD symptoms at study enrollment. Despite setbacks associated with participant recruitment, a low success of enrolling eligible participants and significant attrition during the study, the findings of this small-sample exploratory study uniquely contribute to the limited Zing, online PA literature among Latinx pediatric populations [4,5,11]. Specifically, this study provides rich, real-life considerations for future research on Zing and other online intervention utilization with Latinx youth.

This sample demonstrated variable adherence. Those who demonstrated poor levels of adherence at mid-treatment dropped out shortly after six weeks, while those who demonstrated acceptable levels of adherence at mid-treatment also demonstrated high levels of adherence at post-treatment. Some participants shared that they missed sessions due to family days and religious celebrations, which is not uncommon when working with Latinx populations [34]. Others missed sessions due to prolonged Wi-Fi outages caused by hurricanes, impeding their progress on Zing. These reports were shared with the first author via the weekly adherence summary emails or during the 6-week virtual meeting. At the meeting, parents reported a mix of self-doubt and self-encouragement to continue motivating their child through the intervention, despite the barriers to treatment. Nevertheless, those who demonstrated low adherence at mid-treatment ultimately dropped out shortly after the meeting was held, as evidenced by there being no activity in the Zing platform and a loss of contact. Due to inconsistent adherence rates observed in this study, caused by scheduling conflicts and technological issues, further investigation is needed to evaluate how Zing’s platform compares to traditional clinic-based treatment [10,22,24].

Given the high attrition and low treatment completion rates, the researchers aimed to extract meaningful insights by analyzing all available data regarding cognitive and behavioral outcomes, yielding a statistical between-group and participant-level trajectory analysis. The treatment group demonstrated statistically significant improvements in inattention compared to the WLC group at 6 weeks. Although there is a risk of Type 1 error due to the small sample with this statistic, the finding remains consistent with meta-analytic findings studying coordinative exercise, suggesting that treatment type and duration warrant careful future consideration [5,11]. The individual trajectory analysis revealed that treatment child 2 significantly benefited from the intervention, while the others remained relatively stable in their severity ranges over the course of the condition. There is evidence to support that coordinative exercise improves attentional performance in adolescents [3], and adolescents themselves have credited their improved attention to the Zing program [18]. Until this study, the Zing literature focused on cognitive well-being but had not involved formal measurement of any of the ADHD symptoms assessed in this study [18,19]. While improvements in hyperactivity/impulsivity were not statistically significant, as in prior research [5,6], the results trended in the hypothesized direction as seen in the between-group and participant-level analyses, suggesting preliminary contributions to the emerging Zing literature. It is important to note that Zing seeks to grow from feedback. Thus, the Zing version that was investigated in this current study was slightly different (e.g., visuals) from the one in previous studies and will be slightly different from versions in future investigations but its coordinative exercise core will remain. It is important to note that although our effectiveness inferences are not as robust as they would be if the randomized control trial remained intact, our findings contribute to the broader PA literature by emphasizing the careful attention to measurement that is needed to analyze PA treatment type and duration in marginalized populations [3,10,11].

This study casts a new light on treatment utilization through parent reports of high acceptability. Along with their high acceptability ratings, the parents shared some anecdotal evidence in support of Zing. According to the parent, treatment child 2 demonstrated improvements in concentration on schoolwork and self-control when experiencing high-intensity emotions. Similarly, treatment child 4’s parent reported that both the child and parent enjoyed the program and were able to observe improvements within their everyday routine (e.g., parent–child communication). The high acceptability reports from these two dyads are reassuring because previous findings claim that parents support treatment completion if they believe that it is efficient and effective [4,5,6]. High acceptability in children is crucial for building support for Zing, as the two treatment children demonstrated some enjoyment and some annoyance with the exercises, while previous studies have reported that adolescents found a shortened version of Zing to be enjoyable in a larger sample [18]. It is critical to expand this novel research area to understand the treatment sustainability of various treatment durations and their impact on the Latinx population.

Limitations and Lessons Learned for Future Research. While our exploratory study provides some preliminary findings to support continued examination of Zing, the results should be interpreted with extreme caution. Firstly, recruitment and communication with the target population were barriers to obtaining the proposed sample (i.e., 30) and moving forward with intended data collection and analyses. Therefore, some may argue that limiting inclusion criteria to the Latinx population was restrictive to the participants (*n* = 9) who could have been eligible if ethnicity were not an exclusionary factor. However, it is important to note that because this study focused on the Latinx population, we learned valuable lessons that we otherwise may not have unveiled if this study had a more diverse sample. For example, we learned that similar to Haack et al. [34], recruitment rates improved when the flyers were modified to describe positive connotation on impairment (i.e., potential improvement in attention) rather than targeting symptomology (i.e., inattention). Further, recruitment in this population may require longer timelines compared to samples without a target population. Therefore, researchers should account for an extended timeline when considering an experimental study dependent on research expectations (e.g., master’s thesis, dissertation). In addition, to mitigate poor communication (i.e., lack of responses from parents) with this sample, the researchers used Calendly to streamline scheduling, which improved enrollment rates. Notably, this study sparked interest in Latinx parents who worked as mental health professionals (e.g., therapists, behavior specialists) for themselves and their community, perhaps increasing research interest and participation over time. Even though these parents or their children did not meet the inclusion criteria (e.g., because of age, language, race/ethnicity, symptomology levels), some still shared the flyer with their community. Some mental health professionals and interested parents followed up with the first author to request flyers and study information in Spanish. Therefore, to enhance knowledge in future studies, researchers and intervention developers should implement bilingual materials to include Spanish-speaking Latinx populations.

The small sample hinders generalizability and limits the interpretation of effectiveness and acceptability data. PA intervention studies typically include at least 10 participants when assessing treatment effects [5,6]. In contrast, although our sample started with 13 participants, attrition affected the pre-/post-treatment and pre-/mid-treatment analyses, which included only six and eleven participants, severely impacting the external validity of our study. The inconsistency in sample size across time compromised the robustness of our population-level inferences, significantly limiting the generalizability of our findings to the target population. Fortunately, a crucial strength of our study was that the first author established a strong rapport with the parents, evidenced by the parents sharing their overall impressions with the researcher. The positive impact of carrying out the full study, despite the low sample size and high attrition, was evident at the post-treatment meeting. The two parents who completed the treatment were very thankful that this research was being conducted, especially given their previous efforts in seeking alternative treatments to medication. As the mother of treatment child 2 was providing the researcher with treatment perception, she included improved attention/focus examples (e.g., homework time completion). There is a risk that parental bias is present in the informal results and discussion. Nevertheless, during the meeting with treatment child 2’s mother, her husband verbalized in the background that he agreed with the positive outcomes that the mother was sharing, unprompted, adding more merit to the informal parent report. However, the quantitative data for treatment child 2 demonstrated little to no changes in cognition and behavioral outcomes. Therefore, the addition of a cultural measure (e.g., familism) could have supported the qualitative reporting of real symptomology change, as the two constructs have been suggested to be closely linked within the Latinx population [35]. Future studies should integrate two reporters (e.g., two parents, teachers), depending on the family’s dynamic and their exposure to the child’s daily behavior, to reliably track progress across settings. The differences in perceived outcomes could have been attributed to confounding factors such as socioeconomic status (SES), familism or gender roles because these play a critical role in Latinx outcomes, which also served as a limitation of our study because this data was not collected [34]. Other confounding data that were not collected on concomitant treatments serve as a limitation due to their threat to internal validity.

However, this information came from informal conversations with the researcher and not from qualitative interviews. Qualitative interviews can provide rich insight into a participant’s experience with an intervention, which is critical for identifying necessary culturally relevant components (e.g., language) that are necessary for Latinx communities, an area currently underrepresented in the literature. To better understand Latinx youth experiences with PA intervention, researchers should implement evidence-based recruitment strategies and investigate their participants’ perceptions of treatment utilization and completion with a mixed methods design [4,36].

Lastly, the parent-report nature of the measures is a limitation. Social desirability and expectancy bias can affect the way parents rate their child’s behavior and the intervention acceptability [35], resulting in potentially biased scores. Although informal reports from the parents aided interpretation, our method relied on quantitative progress. For example, although Child 2’s parent provided anecdotal support for her daughter’s improved attention post-treatment, there was no change in the parent-reported quantitative measures from pre- to post-treatment. Moreover, the same parent also expressed that she was proud that the first author was a Latina in higher education. Since ethnoracial concordance is known to play a crucial role for Latinx populations [36], this potentially influenced the high treatment acceptability rating, considering little to no change in their effectiveness outcomes. To combat this limitation, future studies should include multiple reporters (i.e., self and teacher) and conduct qualitative interviews to contextualize the outcomes across respondents and data points, due to the limited state of cultural quantitative measures for cognition and behavioral outcomes.

## 5. Conclusions

This research study represents an important first step in exploring how Latinx families engage with and may benefit from Zing or coordinative exercise. The exploratory findings of Zing are rich and should encourage further investigation into its impact on adherence, cognitive and behavioral outcomes and acceptability within a larger sample of Latinx youth. Informal qualitative data were extremely useful in informing study modifications and encouraging study enrollment and completion for this sample. Future research should incorporate culturally relevant recruitment strategies, measures and qualitative interviews to enrich Latinx research participation and deepen understanding of how updated versions of Zing may support symptom improvement in Latinx youth.

## Figures and Tables

**Table 1:** Participant demographics.

			Randomized (*N* = 13)		Completed Half of T (*N* = 11)		Completed T (*N* = 6)	
			T (*n* = 6)	WLC (*n* = 7)	T (*n* = 5)	WLC (*n* = 6)	T (*n* = 2)	WLC (*n* = 4)
			M (SD)	M (SD)	M (SD)	M (SD)	M (SD)	M (SD)
Child								
	Age (years)		8.2 (1.5)	8.3 (1.4)	8.2 (1.6)	8 (1.3)	8.5 (2.1)	8.5 (1.3)
	Girl/Female *n*		2	1	1	2	1	2
	Boy/Male *n*		4	4	4	4	1	2
Child Ethnicity								
	Latinx ethnicity of any race		5	6	4	5	1	3
	White, Latinx ethnicity of any race		1	1	1	1	1	1
Parent								
	Relationship *n*							
	Biological Mother		6	6	5	4	2	3
	Adoptive Mother		-	1	-	1	-	1

Note: T = Treatment, WLC = Waitlist Control, M = Mean, SD = Standard Deviation.

**Table 2:** Mann–Whitney test statistic for pre- and mid- timepoints (N = 11).

Variable	Mann–Whitney Test Statistic	*p*-Value
Working Memory	6	0.06
Inhibition	6.5	0.07
Inattention	0	0.004 *
Hyperactivity/Impulsivity	6.5	0.07

* *p* < 0.005.

## Data Availability

The original data contribution from the current study are included in the article; further inquiries can be directed to the corresponding author.
